# Editorial: The clinical utility of long read sequencing to improve diagnostic yield and uncover biological mechanisms in rare disease

**DOI:** 10.3389/fgene.2024.1494860

**Published:** 2024-10-04

**Authors:** Lidia Larizza, Christopher M. Watson, Madelyn A. Gillentine, Palma Finelli

**Affiliations:** ^1^ Research Laboratory of Medical Cytogenetics and Molecular Genetics, IRCCS Istituto Auxologico Italiano, Milan, Italy; ^2^ Institute of Medical Research, St James’s University Hospital, University of Leeds Faculty of Medicine and Health, Leeds, United Kingdom; ^3^ North East and Yorkshire Genomic Laboratory Hub, Central Lab, St James’s University Hospital, Leeds Teaching Hospitals NHS Trust, Leeds, United Kingdom; ^4^ Department of Laboratories, Seattle Children’s Hospital, Seattle, WA, United States; ^5^ Dipartimento di Biotecnologie Mediche e Medicina Traslazionale, University of Milan, Milan, Italy; ^6^ Medical Genetics Laboratory, Fondazione IRCCS Ca’ Granda Ospedale Maggiore Policlinico, Milan, Italy

**Keywords:** long-read sequencing, genetic diseases, structural variants, chromothripsis, tandem repeat-related diseases, transcriptomics, epigenetic modifications

Long Read Sequencing (LRS), a multi-omics technology impacting genomics, epigenomics, and transcriptomics overcomes the limitations of short read exome/genome sequencing (SRS) and other second generation techniques in disclosing the hidden basis of rare genetic diseases (Mastrorosa et al., 2023; Yu et al., 2023; Kernohan et al., 2024).

**FIGURE 1:**
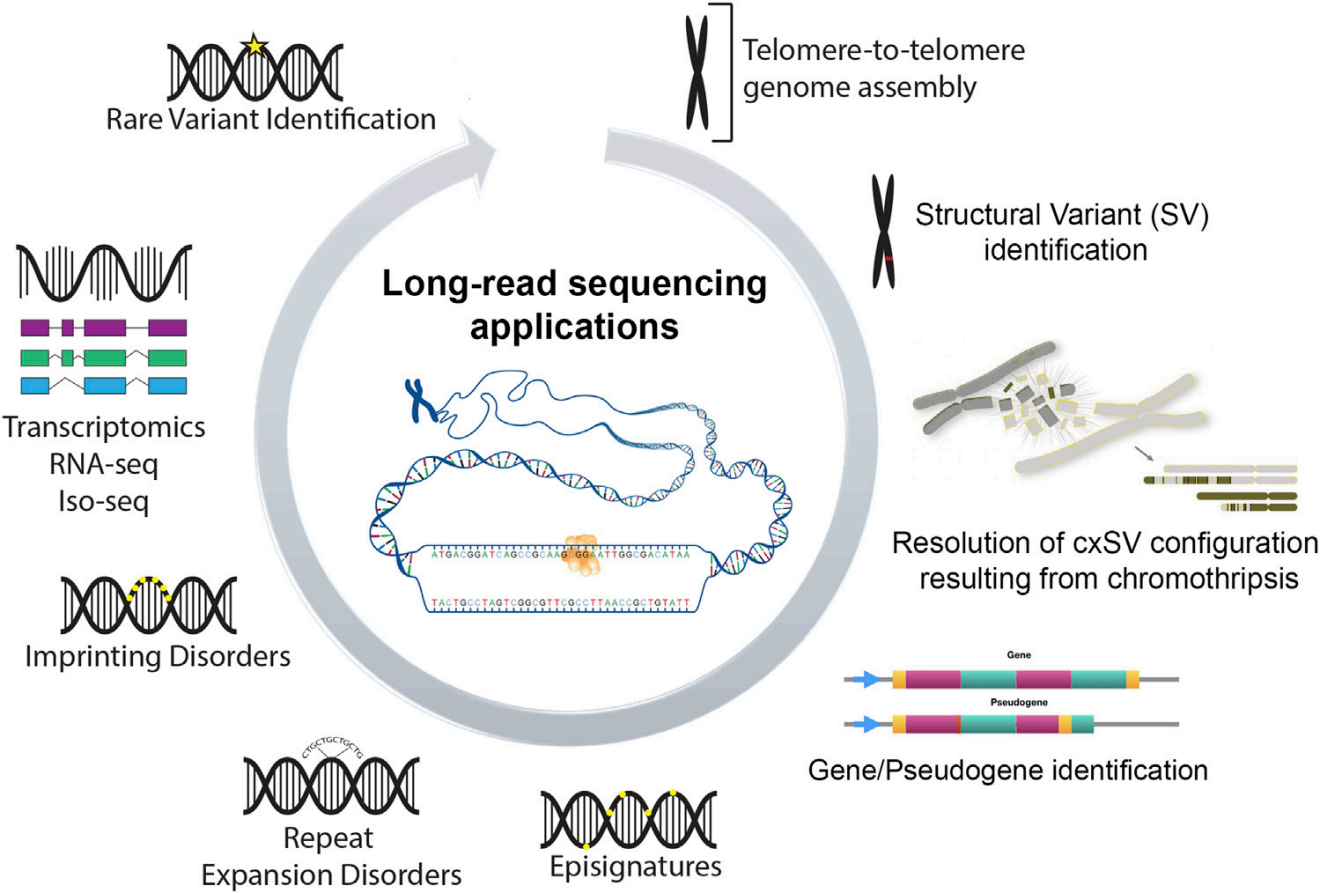
Emerging applications of long-read sequencing.

Figure 1 summarizes emerging applications of LRS data. These include an ability to identify the precise configuration of simple and complex structural variants at nucleotide resolution (such as those originating from chromothripsis), the unambiguous alignment of sequence reads to functional or related pseudogenes loci, characterization of specific diseases episignatures, complete read-through of expansions in tandem repeats disorders, identification of epi-transcriptomic modifications of imprinting disorders and identification of variants in the intronic regions that may result in abnormal splicing and/or of novel transcripts. Such capabilities increase the variable and often unsatisfactory diagnostic yield achieved from SRS assays (25%–50%) (Sullivan et al., 2023). Despite these advancements LRS is nevertheless typically applied in the research context and is yet to be routinely deployed in the clinical setting.

Our themed research collection is a bit of straw to promote the application of LRS in the clinical setting, prioritizing groups of rare diseases in which molecular mechanisms are refractory or hard to identify by second generation sequencing technologies.

The article by Ura et al. addresses the development of target capture full length double-stranded cDNA sequencing by nanopore LRS to uncover intronic variants in the Tuberous Sclerosis type 1 and type 2 (*TSC1* and *TSC2*) genes in a clinically affected but molecularly undiagnosed individual. The occurrence of deep intronic variants generates novel transcripts with intron retention leading to a truncated protein and a decreased potential of full-length isoforms in respect to healthy controls. The Authors define the repertoire (number, coverage, exon number, transcript length) of *TSC1* and *TSC2* transcripts and focus on the “protein coding” transcripts. Reduced expression of such transcripts leads to identification of a *TSC2* variant in the proband, which is then validated by an *in vitro* assay. Besides providing a diagnosis for this individual, the delineated multi-step experimental pathway confers the essential information to monitor the full-length alternative splicing of transcripts for the diagnosis of genetic diseases.

Another limitation of second-generation sequencing workflows, even when considered in combination with high resolution array-based comparative genomic hybridization (a-CGH), is the sensitivity to identify and completely characterize (at single nucleotide resolution complex structural variants.

The article by Bestetti et al. addresses this challenging issue by target Oxford Nanopore sequencing on a proband with a clinical diagnosis of Cornelia de Lange syndrome (CDLS), for whom first-tier testing identified an abnormal karyotype 46, XY, t(5; 15)(p13; q25) dn. FISH analyses mapped the putative translocation breakpoints on der(5) within intron 2 of *ADAMTS12* gene- 3 Mb from the *NIPBL* 5’UTR- and on der(15) within intron 1 of *SEMA4B*. While the reduced expression of the *NIPBL* transcript, from exon 23 to 3’UTR accounted for the clinical phenotype, only LRS unraveled the configuration and origin of the cryptic complex structural variant (cxSV). Besides confirming the previous mapping on derivative chromosomes, analysis of nanopore-generated sequence reads showed the signature of a previous chromothripsis event on der(5) leading to the shattering at 5p13.2 of a 7.3 Mb region, comprising 44 coding genes, into 17 fragments relocated in a random order and orientation, with 36 underlying breaks. Despite the large number of coding genes, the “all at once” rearrangement on der(5) disrupted only 3 genes with a single break in *ADAMTS12* and *C6* and 16 breaks in *NIPBL*. Notably *NIPBL* was the main target with 16 breaks clustering between introns 21 and 41, several coinciding with repeated SINE and LINE elements and a segmental duplication at intron 21, suggesting proneness to rearrangement of this unstable region. A single breakpoint was identified on der(15) where the juxtaposition between the short arm of chromosome 5 and the long arm of chromosome 15 led to a fusion gene between *SEMA4B* (5’UTR-intron1) and *ADAMTS12* (intron 2–3’ UTR), not contributing to the clinical phenotype as not transcribed. In conclusion the *NIPBL* gene, accounting for 50%–60% of CDLS cases, is worth assessment by LRS to unravel gross rearrangements in clinically suspected, molecularly undiagnosed cases.

The review article by Olivucci et al. provides a comprehensive and critical overview of the relevant advantages of LRS to the diagnosis of rare genetic diseases predicting, as suggested by the title, a trend towards its application in the clinical context. Given the capability of LRS technologies to sequence long molecules of nucleic acids (10–100 Kb and longer) resulting in improved mappability and enabling the evaluation of different classes of variation in a single analysis, the Authors review SRS shortcomings that are overcome by LRS. A considerable challenge for SRS are structural variants (SVs), that range from 50 bps to megabases in length and include cxSVs. The Authors emphasise that the precise identification of SV breakpoints remains one of the most important clinical applications of LRS and discuss a range of genetic disorders, including cancer susceptibility syndromes, where resolved SV and cxSV (Scharf et al., 2022) demonstrate the potential of LRS as a powerful genetic tool in the hereditary cancer setting. As to tandem repeat (TR)-related diseases, another SRS limitation, the Authors remark the contribution of LRS to discovery of novel disease-associated TRs. The combination of CRISPR-Cas9 enrichment and LRS outperformed conventional techniques in accurate determination of repeat length and interruptions, even in mosaic alleles. However, improvements in terms of diagnostic yields and time to diagnosis are needed to make LRS the first tier-test for the diagnosis of TR-diseases. An additional advantage of LRS is accessing difficult to sequence repetitive or duplicated regions, such as those containing disease-associated genes highly homologous to pseudogenes (e.g. *PKD1*, *CYP21A2*, *SMN1*). Targeted LRS ensures high coverage and a reduction in alignment errors (caused by short sequence reads), facilitating the identification of single nucleotide variants (SNVs) or SVs. As to the diagnosis of imprinting disorders and identification of episignatures for novel genetic disorders, the Authors believe LRS currently has a limited impact: they devote the final section to transcriptome analysis recommending to assess both mis-spliced transcripts and altered isoforms in the workflow for molecular genetic diagnosis.

The article by Wang et al. reports that out of 21,840 Chinese newborns with suspected inborn errors of metabolism (IEMs) hospitalized from 2017 to 2022, 3,211 had confirmed the clinical diagnosis by tandem mass-spectrometry, 111 of which underwent genetic testing by whole exome sequencing which disclosed pathogenic variants in 49 cases. One wonders whether LRS approaches on WES-negative patients and further IEMs cases investigated in the last years might complete the diagnostic flowchart of this relevant project.

Overall, the manuscripts in this Research Topic highlight some of the ways that LRS can advance diagnostic testing in multiple disease areas, including cancer and rare disease.
